# UNMET NEEDS FOR MENTAL HEALTH CARE: ARE THERE RACIAL DISPARITIES AMONG OLDER AMERICANS IN NEW YORK?

**DOI:** 10.1093/geroni/igae098.2465

**Published:** 2024-12-31

**Authors:** Chien-Ching Li, Pei-Shan Yen, Yi-Fan Chen, Alicia Matthews

**Affiliations:** Rush University, Chicago, Illinois, United States; University of Illinois Chicago, Chicago, Illinois, United States; University of Pittsburgh, Pittsburgh, Pennsylvania, United States; Columbia University, New York, New York, United States

## Abstract

Racial and ethnic differences in mental health problems have been widely discussed in the existing literature. However, limited research examines the disparities in mental health care among older Americans, who have been found to face access barriers to care. This study aimed to examine the racial and ethnic disparities in unmet needs for mental health care among older Americans in New York. We analyzed data from the New York City Community Health Survey (NYC CHS; 2012-2020). Our study sample included participants aged 50 years and older from the NYC CHS. The dependent variable of interest was the unmet need for mental health care (yes, no), which was assessed through the question, “Did you need treatment for mental health but did not receive it?”. The independent variable was race/ethnicity (non-Hispanic White, non-Hispanic Black, Hispanic, non-Hispanic Asian/Pacific Islander, other non-Hispanic). Covariates measured at a multi-level, including demographics, access to care, and neighborhood factors, were included. Regression results indicated racial/ethnic disparities in unmet needs for mental health care. Individuals in other non-Hispanic group were more likely to report having unmet needs for mental health care. However, non-Hispanic Blacks and Asian/Pacific Islanders exhibited a higher likelihood of having unmet mental health care needs compared to Whites. More importantly, older individuals without a personal doctor and those residing in underprivileged neighborhoods were associated with a higher likelihood of having unmet mental health care needs. Further development of community-based interventions is needed to mitigate access barriers to mental health care among older Americans.

